# Ebselen oxide and derivatives are new allosteric HER2 inhibitors for HER2‐positive cancers

**DOI:** 10.1002/1878-0261.13419

**Published:** 2023-03-27

**Authors:** Lucas Blasquez, Haniaa Bouzinba‐Segard, Sandrine Bourdoulous, Camille Faure

**Affiliations:** ^1^ Université Paris Cité, CNRS, INSERM, Institut Cochin Paris France

**Keywords:** additive effects, breast cancer, HER2 tyrosine kinase receptor, resistance, small molecule inhibitor, targeted therapy

## Abstract

Human epidermal growth factor receptor 2 (ErbB2/HER2) is a tyrosine kinase receptor that is overexpressed in 25% of primary human breast cancers, as well as in multiple other cancers. HER2‐targeted therapies improved progression‐free and overall survival in patients with HER2^+^ breast cancers. However, associated resistance mechanisms and toxicity highlight the need for new therapeutic approaches for these cancers. We recently established that, in normal cells, HER2 is stabilized in a catalytically repressed state by direct interaction with members of the ezrin/radixin/moesin (ERM) family. In HER2‐overexpressing tumors, the low expression of moesin contributes to the aberrant activation of HER2. Through a screen designed to find moesin‐mimicking compounds, we identified ebselen oxide. We show that ebselen oxide, and some derivatives, conferred an efficient allosteric inhibition of overexpressed HER2, as well as mutated and truncated oncogenic forms of HER2, which are resistant to current therapies. Ebselen oxide selectively inhibited anchorage‐dependent and ‐independent proliferation of HER2^+^ cancer cells and showed a significant benefit in combination with current anti‐HER2 therapeutic agents. Finally, ebselen oxide significantly blocked HER2^+^ breast tumor progression *in vivo*. Collectively, these data provide evidence that ebselen oxide is a newly identified allosteric inhibitor of HER2 to be considered for therapeutic intervention on HER2^+^ cancers.

## Graphical abstract

The tyrosine kinase receptor HER2 is a major oncogene in multiple cancers. We identified ebselen oxide as a new allosteric inhibitor of HER2. Ebselen oxide maintains HER2 in a catalytically repressed state by targeting the ezrin/radixin/moesin (ERM)‐binding motif in the juxtamembrane region of HER2. Ebselen oxide reduced the progression of HER2‐positive tumors and showed a significant benefit in combination with current HER2‐targeted therapies.

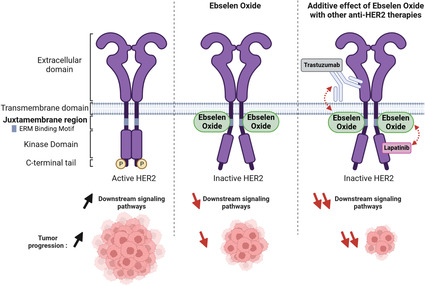

**Abbreviations**
Aa: amino acid
Akt: protein kinase B
CF3: trifluoromethyl
CH3: methyl group
Cl: chloride
DMEM: Dulbecco's Modified Eagle Medium
EBM: ERM‐binding motif
Ebs ox: ebselen oxide
EGF: epidermal growth factor
EGFR/HER1: epidermal growth factor receptor‐1
ErbB2: erythroblastic oncogene B2
ERK: extracellular signal‐regulated kinase
ERM: ezrin/radixin/moesin
FBS: fetal bovine serum
FERM: band 4.1, ezrin, radixin, moesin
GST: glutathione S‐transferase
H: hydrogen
HBMECs: human bone marrow endothelial cells
HER2: epidermal growth factor receptor‐2
HRG: heregulin
JM_HER2_
: HER2 juxtamembrane region
Lapat: lapatinib
MAPK: mitogen‐activated protein kinase
OMe: methoxy group
PI3‐Kinase: phosphatidylinositol‐3 kinase
S: sulfur
Se=O: selenium oxide
SPR: surface plasmon resonance
Trastu: trastuzumab

## 1 Introduction

ErbB2/HER2 (human epidermal growth factor receptor 2) is an oncogenic tyrosine kinase receptor overexpressed in 25% of primary human breast cancer cases as a result of *ErbB2* gene amplification or enhanced expression. HER2 overexpression is associated with higher rates of disease recurrence and mortality. In addition, HER2^+^ breast cancers have a higher predilection to metastasize to the brain [1]. Treatment of HER2^+^ breast cancers relies on HER2‐targeted therapies consisting of monoclonal antibodies (trastuzumab, pertuzumab) or kinase inhibitors (lapatinib). These targeted agents had brought a robust clinical benefit for a subset of patients with HER2^+^ breast cancers. However, the majority of these patients will experience disease progression owing to primary and acquired resistance [2, 3]. Among resistance mechanisms are the expressions of a truncated form of HER2 (p95HER2) that lacks the extracellular domain targeted by trastuzumab, or expression of HER2 with mutations in the kinase domain, such as V777L or V842I, responsible for constitutive kinase activity [4, 5, 6]. The use of kinase inhibitors is also associated with strong adverse effects as a result of nonspecific targeting of non‐HER2 kinases [7]. This highlights the need to develop new therapeutic approaches to overcome these limitations and improve the clinical outcome of HER2^+^ breast cancer patients. More recently, HER2 has also been established as an important target in multiple cancers, including gastric, ovarian, pancreatic, and kidney cancers as a consequence of its gene amplification or the presence of somatic mutations, driving HER2 activation and tumor aggressiveness [8, 9]. Hence, developing new HER2‐directed therapeutic agents is important to improve the clinical management of several HER2^+^ cancers.

HER2 is a transmembrane tyrosine kinase receptor, which belongs to the HER family: HER1/EGFR (epidermal growth factor receptor), HER2 (neu, c‐erbB2), HER3, and HER4 [10]. HER2 plays important roles in various cellular processes, such as proliferation and differentiation, and is a key regulator of cardiac function [11]. Among the large family of tyrosine kinase receptors, HER2 displays unusual structural features conferring unique properties. Structurally, HER2 contains an extracellular domain locked in an open conformation [12], a transmembrane domain, a cytosolic juxtamembrane region, a tyrosine kinase domain, and a C‐terminal tail containing regulatory tyrosine residues. HER2 has no known ligand and despite its structural particularity, HER2 remains inactive, unless overexpressed or activated in heterodimers with the other HER family members upon cognate ligand stimulation [10]. Heterodimer formation is driven by receptor availability, as well as the nature of the ligand. For instance, EGF stimulation favors the formation of EGFR/HER2 heterodimers, while Heregulin (HRG) promotes ErbB3/HER2 heterodimers [13]. Ligand binding results in the activation of the dimers and autophosphorylation of the tyrosine residues located in the C‐terminal tail triggering the activation of two main signaling pathways, PI3‐Kinase/Akt and MAPK ERK pathways controlling cell survival and proliferation, respectively. Moreover, we recently demonstrated that, unlike its family members, HER2 possesses in its cytosolic juxtamembrane region (aa 676–691) a binding motif for the ERM (ezrin, radixin, and moesin) proteins, known to link transmembrane proteins to the cortical actin cytoskeleton, thus controlling receptor localization, surface availability and signaling [14, 15]. We showed that ERM/HER2 interaction stabilizes HER2 in a catalytically repressed state. In addition, we found a highly significant inverse correlation between moesin expression and HER2 status in primary tumors of different cancers, including breast, cervix, stomach, bladder, pancreas, colon, ovary, and kidney. Low moesin expression was correlated with enhanced aberrant ligand‐independent activation of HER2, which could be reverted by restoring the expression of ERM proteins. Through a high‐throughput assay based on HER2/ERM interaction to find moesin‐mimicking compounds, we previously identified zuclopenthixol that behaved as moesin in maintaining HER2 in a catalytically repressed state, hence providing a novel therapeutic approach targeting HER2^+^ breast cancers and brain metastasis [14].

Here, we demonstrate that another compound that came out from the screen, ebselen oxide, and some of its derivatives, also efficiently blocked HER2 activation by interacting with the ERM‐binding motif contained in the cytosolic juxtamembrane region of HER2. Ebselen oxide inhibited the proliferation and the anchorage‐independent growth of HER2^+^ breast, gastric, and ovarian cancer cells *in vitro*. It also strongly inhibited HER2 activation and HER2‐dependent proliferation of cells expressing truncated and mutated forms of HER2. Ebselen oxide administration strongly decreased HER2 signaling and tumor growth of breast cancer cells orthotopically implanted in the mammary fat pad of immunodeficient mice. Finally, we provide evidence that combining ebselen oxide with trastuzumab or lapatinib strongly enhanced their antitumoral effect in HER2^+^ cancer cells. These results reveal that ebselen oxide is a novel allosteric inhibitor of HER2, which behaves as a moesin‐mimicking compound, and which may improve the treatment of HER2‐dependent tumors.

## 2 Materials and methods

### 2.1 Reagents

Antibodies used were as followed: polyclonal anti‐pY1248 HER2 (ref 06–229, WB‐1 : 1000) was purchased from Merck Millipore (Darmstadt, Germany), polyclonal anti‐HER2 (ab2428, WB‐1 : 1000), and polyclonal anti‐β‐tubulin (ab15568, WB‐1 : 1000) were purchased from Abcam. Monoclonal anti‐HER2 (clone Cle2‐4001 and 3B5, IF‐1 : 100) and polyclonal anti‐KI67 (ref PA5‐16785, IF‐1 : 200) were purchased from Thermo Scientific (Waltham, MA, USA). Monoclonal anti‐pERK (pErk1/2 Thr202/Tyr204, ref 9106, WB‐1 : 500), polyclonal anti‐ERK (ref 9102, WB‐1 : 500), polyclonal anti‐pAkt (pAKT Ser473, ref 9271, WB‐1 : 1000), and polyclonal anti‐Akt (clone 9272, WB‐1 : 1000) were purchased from Cell Signaling Technology (Danvers, MA, USA). Polyclonal anti‐ezrin (WB‐1 : 1000) was a kind gift of Dr C. Roy CNRS UMR 5539. Monoclonal antiphosphotyrosine (clone 4G10, WB‐1 : 100) was from Meditech (Waltham, MA, USA). Fluorescence‐conjugated secondary antibodies, peroxidase‐conjugated secondary antibodies, and ECL reagents were from Jackson Immunoresearch Laboratories (Cambridge, UK). DAPI and MTT (3‐(4,5‐dimethylthiazol‐2‐yl)‐2,5‐diphenyltetrazolium bromide) reagents were from Sigma. EGF and Heregulin‐1ß were from BD Biosciences (Franklin Lakes, NJ, USA). The EGFR/HER2 tyrosine kinase inhibitor Tyrphostin AG1478 was from Merck Sigma (Darmstadt, Germany). Lapatinib ditosylate was from Santa Cruz Biotechnology (Dallas, TX, USA) and trastuzumab (Ontruzant) was from Samsung Bioepis (Songdo, South Korea). Ebselen, ebselen oxide and derivatives [analog 1 (2‐(4‐methylphenyl)‐1,2‐benzisothiazol‐3(2H)‐one), analog 2 (2‐(4‐chlorophenyl)‐1,2‐benzisothiazol‐3(2H)‐one), analog 3 (2‐(4‐methoxyphenyl)‐1,2‐benzoselenazol‐3‐one), analog 4 (N‐phenylphtalimide), analog 5 (2‐(4‐isopropyl phenyl)‐1‐isoindolinone), analog 6 (2‐(4‐Methylphenyl)‐1‐oxo‐1,2‐benzothiazol‐3‐one), analog 7 (2‐[4‐(trifluoromethyl)phenyl]‐1,2‐benzothiazol‐3‐one), and analog 8 (2‐(4‐chlorophenyl)‐1‐oxo‐1,2‐benzothiazol‐3‐one)] were synthesized and purified by Roowin (Riom, France).

### 2.2 Alpha screen assays

Biotinylated peptide derived from HER2 cytosolic juxtamembrane region (JM_HER2_, ILIKRRQQKIRKYTMRRL) has been synthesized by Proteogenix (Schiltigheim, France). The binding reaction was described previously [14]. Briefly, white 384‐well Optiplates (PerkinElmer, Whalham, MA, USA) were used to prepare GST‐FERM_E_ and biotinylated peptide in a binding buffer (PBS, pH 7.4, 5 mm MgCl_2_, and 0.02% CHAPS). GST‐FERM_E_ was incubated with biotinylated‐HER2 for 30 min at room temperature. AlphaScreen^®^ Streptavidin Donor beads (20 μg·mL^−1^) and Glutathione AlphaLISA^®^ Acceptor Beads (20 μg·mL^−1^) were added to the wells for overnight incubation in the dark and at room temperature. Light signal was detected with the EnVision^®^ multilabel plate reader (PerkinElmer).

### 2.3 Surface Plasmon resonance

Interaction was performed on a BIAcore T200 instrument BIAcore (Cytiva Life Sciences, Marlborough, MA, USA); GE Healthcare (Little Chalfont, UK). Streptavidin‐coated sensor chip (Series S sensor chip SA, GE Healthcare) was used to capture peptide derived from the cytosolic juxtamembrane sequence of HER2 (surface immobilization level of 401 RU) at a flow rate of 5 μL·min^−1^ in a running buffer (10 mm Hepes, 150 mm NaCl, 1 mm EDTA, 1 mm DTT, 0.005% Tween20). Ebselen or ebselen oxide was used as an analyte and injected at 5 or 10 μm at a flow rate of 25 μL·min^−1^ for 60 s.

### 2.4 Cell culture conditions

Human bone marrow endothelial cells (HBMECs) kindly provided by Dr. B. Weksler (Weill Medical College of Cornell University, NY) were cultured as described previously [16]. The human breast cancer cell lines [SKBR3 (RRID:CVCL_0033), BT474 (RRID:CVCL_0179), and MDA‐MB‐231 (RRID:CVCL_0062)], the human gastric [NCI‐87 (RRID:CVCL_1603)] and ovarian [SKOV3 (RRID:CVCL_0532)] cancer cell lines were obtained from the American Type Culture Collection (Manassas, VA) and were maintained in DMEM containing 10% FBS. All cell lines were cultured under 5% CO2 at 37 °C. HBMECs were transfected using Amaxa Inc nucleofector system (Kit V and U015 program). FBS was decreased to 1% to analyze HER2 activation status and HER2‐dependent cell proliferation in SKOV3 as previously described [17]. Periodic tests for Mycoplasma and authentication were performed using commercially available kits.

### 2.5 Western blots

Cells were washed with cold PBS and lysed in a buffer containing 1% Triton, 50 mm Tris–HCl pH 8.8, 25 mm NaCl, 1 mm EDTA, 1 mm Na_3_VO_4_, 1 mm AEBSF, 10 μg·mL^−1^ each of aprotinin/leupeptin/pepstatin or lysed in Laemmli buffer. Proteins were separated by SDS/PAGE and transferred to nitrocellulose (Schleicher & Schuell, Keene, NH, USA). Membranes were blocked for 1 h in TBS/0.1% Tween20/3% BSA and probed overnight with indicated antibodies. Peroxidase‐coupled secondary antibodies were used to reveal antibody binding using the ECL substrate (Pierce, Thermo Fisher Scientific, Waltham, MA, USA) and Fusion FX (Vilber Lourmat, Collégien, France) chemoluminescence acquisition system. Quantifications were done with imagej software (1.53e) (NIH, USA).

### 2.6 Plasmid and mutagenesis

Vector encoding wild‐type glycoprotein D‐tagged form of HER2 (gD‐HER2‐WT) [18] was a kind gift of Dr M. Sliwkowski (Genentech, San Francisco, CA, USA). The gD‐tagged HER2 mutant (gD‐HER2‐EBM*) and point mutations in the kinase domain (V777L and V842I) were previously described [14]. Vector encoding the HER2p95 [19] was provided by Dr J. Baselga.

### 2.7 Proliferation assays

#### 2.7.1 MTT

Cell medium was removed and replaced with fresh culture medium. 3‐(4,5‐dimethylthiazol‐2‐yl)‐2,5‐diphenyltetrazolium bromide (MTT reagent) was added at a final concentration of 1.2 mm for 2–4 h at 37 °C. Produced formazan was solubilized with DMSO and measured by 540 nm absorbance reading.

#### 2.7.2 xCELLigence

9.10^3^/96‐well (BT474 or SKBR3) cells or 6.10^3^/96‐well (MDA‐MB‐231) cells were seeded on E‐Plate 96 (ACEA, Agilent, Santa Clara, CA, USA), treated as indicated and impedance was measured in real‐time using an RTCA MP‐Station.

#### 2.7.3 Incucyte

25.10^3^ cells/96‐well transfected HBMECs or 9.10^3^ cancer cells were seeded and monitored with an Incucyte S3 imaging system (Sartorius, Goettingen, Germany) using 10× objective every 2 h during 5 days. Confluency analyses were performed using the dedicated Incucyte analysis system.

### 2.8 Anchorage‐independent growth assay

#### 2.8.1 Soft agar assay

A bottom layer of 0.8% agarose in DMEM supplemented with 20% FBS and penicillin/streptomycin was added to 24‐well plates before seeding 25.10^3^ cells per well in a 0.6% agarose top layer. Cells were treated with a vehicle or with 10 μm ebselen oxide or derivatives. Treatments were renewed 3 times a week. After 6 weeks, colonies were visualized with a phase‐contrast microscope and their number and size were quantified using imagej software.

#### 2.8.2 Ultra‐low attachment (ULA) plating

5.10^3^ cells/24‐well were seeded in a complete medium in ULA plates (Corning^®^ Costar^®^ (Corning, NY, USA) C LS3473‐24EA) in the presence of indicated compounds. Spheroids were imaged 6–8 days later with a phase‐contrast microscope, and their areas were measured using imagej software.

### 2.9 Tumor growth in orthotopic xenograft

Experiments were done as already described [14] on the TrGET platform of centre de Recherche de Cancérologie de Marseille (Institut Paoli Calmettes, Marseille, France) in accordance with the guidelines of the Institut National de la Santé et de la Recherche Médicale. The study was approved by the Animal Experimentation Ethics Committee of Marseille (APAFIS#2079‐2015092811101360 v3). Briefly, 7‐to‐8‐week‐old female NOD.Cg‐Prkdc scid/J (NSG) mice were purchased from Charles River France or bred in‐house and maintained on a 12‐h light and 12‐h dark cycle, under specific pathogen‐free conditions with sterilized food and water provided *ad libitum*. The initial body weight of the animals at the time of arrival was between 19 and 24 g. Mice were allowed to acclimatize to local conditions for 1 week before being injected with tumor cells. 5 × 10^6^ BT474 cells resuspended in 100 μL (50% matrigel and 50% PBS) were implanted orthotopically in the mammary fat pad of NSG mice anesthetized with ketamine and xylazine and administered with the nonsteroidal anti‐inflammatory drug metacam (SC, 1 mg·kg^−1^). Estradiol pellets were implanted subcutaneously. Metacam was administered 24 and 48 h postimplantation. Nineteen days after implantation, mice were randomized into 2 groups to be administered intraperitoneally during 3 weeks with vehicle (10% DMSO in PBS, twice a day, 5 days a week, *n* = 8), 3 mg·kg^−1^ ebselen oxide (twice a day, 5 days a week, *n* = 8), or 5 m·kg^−1^ ebselen oxide (twice a day, 5 days a week, *n* = 8). Mice weight and tumor volume were assessed 2–3 times a week using a caliper. At the end of the experiment, the collected tumors were photographed, weighed, and cut into halves to be embedded in OCT for immunohistofluorescence analysis or frozen in liquid nitrogen for western blot analysis. A tissue Lyzer™ was used to solubilize proteins in a buffer containing 50 mm Tris pH = 8.8, 25 mm NaCl, 1 mm EDTA, 1% Triton, 10% glycerol, and 1 mm phosphatase (AEBSF and 1 mm orthovanadate), and 10 μg·mL^−1^ protease inhibitors (aprotinin, leupeptin, pepstatin).

### 2.10 Immunofluorescence analysis of tumor sections

The collected tumors embedded in OCT were cut into 5 μm thick sections and immobilized on superfrost™ plus microscope slides. Sections were fixed in 4% paraformaldehyde for 10 min at room temperature, washed three times in PBS, permeabilized for 10 min in PBS containing 0.1% Triton X100, washed with PBS, blocked for 30 min in PBS containing 3% BSA and incubated 1 h at room temperature with primary antibodies [Monoclonal anti‐HER2 (clone Cle2‐4001 and 3B5, IF‐1 : 100) or polyclonal anti‐KI67 (ref PA5‐16785, IF‐1 : 200)]. Sections were again washed three times in PBS and incubated 1 h at room temperature with fluorescence‐conjugated secondary antibodies from Jackson Immunoresearch Laboratories. After mounting, samples were scanned using a Lamina multilabel slide scanner (PerkinElmer). The proliferative index (mean percentage of Ki67‐positive nuclei) was quantified on 10 regions (area 0.2 mm^2^) per slide (vehicle, *n* = 7; ebselen oxide *n* = 5) using imagej software.

### 2.11 Statistical analyses

Statistical analyses were performed by graphpad prism 5 software (La Jolla, CA, USA). The Student *t* test was used to analyze the statistical difference between the two groups. The one‐ or two‐way ANOVA tests followed by the multiple comparison test, as indicated, were used to analyze the statistical difference between multiple groups. Data are presented as the mean ± SEM as indicated. *P* value < 0.05 was considered statistically significant.

## 3 Results

### 3.1 Ebselen oxide is a moesin‐mimicking compound conferring allosteric inhibition of HER2

**Fig. 1:**
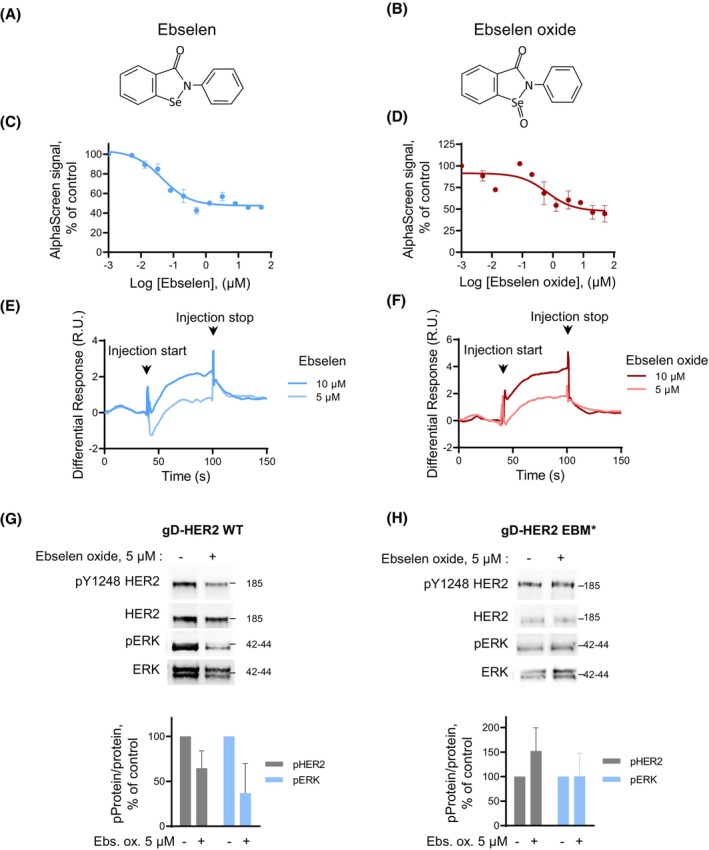
Ebselen oxide inhibits HER2 activation through binding to the ERM‐binding motif in the cytosolic juxtamembrane region of HER2. (A, B) Chemical structures of ebselen (A) and ebselen oxide (B). (C, D) Dose‐dependent inhibition of the interaction between the biotinylated peptide coding for the cytosolic juxtamembrane regions of HER2 (biot‐JM_HER2_, 5 nm) and the N‐terminal FERM domain of ezrin (GST‐FERM_E_, 156 μm) in the presence of ebselen (C) or ebselen oxide (D) at increasing concentrations (0–50 μm) for 20 h measured by AlphaScreen®. A representative experiment is shown where data are mean ± sem (*n* = 2) out of 3 (C) or 2 (D) independent experiments. (E, F) Sensorgrams showing binding of ebselen (E) and ebselen oxide (F) to biot‐JM_HER2_ immobilized on a streptavidin‐coated sensor chip (surface immobilization level of 401 RU). Arrows indicate the beginning and the end of the injection. A representative experiment is shown out of 3 (E) or 2 (F) independent experiments. (G, H) Lysates from HBMECs transfected with vectors encoding gD‐tagged wild‐type HER2 (gD‐HER2‐WT) (G) or the mutant generated with alanine and glycine substitutions within the ERM‐binding motif (gD‐HER2‐EBM*) (H) and treated or not during 24 h with 5 μm ebselen oxide were analyzed by western blot using antibodies against activated HER2 (pY1248), HER2, activated ERK (pERK), and ERK. Representative blots and quantification of data are shown where data are mean ± sem [*n* = 3 (G); *n* = 2 (H)].

We previously performed a high‐content screen designed to identify a moesin‐mimicking compound interacting with the ERM‐binding motif contained in the cytosolic juxtamembrane region of HER2. Ebselen came out as an interesting hit [14]. Ebselen is a synthetic organoselenium compound with the anti‐inflammatory and antioxidant property that has been evaluated in clinical trials for ischemic stroke and acute hearing loss [20, 21]. However, *in vivo*, ebselen rapidly reacts with peroxynitrite to form ebselen oxide [22]. We therefore analyzed the effect of both ebselen and ebselen oxide *in vitro* (Fig. 1A,B), on the interaction between a biotinylated peptide derived from the cytosolic juxtamembrane region of HER2 (JM_HER2_) and the N‐terminal FERM domain of ezrin (FERM_E_) using a protein–protein interaction assay (AlphaScreen), and on HER2 activation, as described previously [14]. Ebselen and ebselen oxide both dose‐dependently competed with JM_HER2_/FERM_E_ interaction reaching, respectively, 53% and 52% inhibition at 20 h for ebselen (IC_50_ = 44 nm) and ebselen oxide (IC_50_ = 634 nm) (Table 1 and Fig. 1C,D). In addition, using surface plasmon resonance (SPR), we confirmed that both compounds could directly bind to JM_HER2_ (Fig. 1E,F). However, while ebselen poorly inhibited HER2 activation (Fig. S1), treatment with 5 μm ebselen oxide of human endothelial cells (HBMEC) ectopically overexpressing a glycoprotein D‐tagged form of wild‐type HER2 (gD‐HER2‐WT) reduced by 40% the phosphorylation level of gD‐HER2‐WT, accompanied by a 65% reduction in the activation of ERK protein kinase (Fig. 1G). The slight decrease in HER2 expression was not significant. To examine whether HER2 inhibition by ebselen oxide was dependent on JM_HER2_/FERM_E_ interaction, we transfected HBMEC cells with a previously described glycoprotein D‐tagged HER2 form mutated on the critical amino acids involved in ERM binding (gD‐HER2‐EBM*) [14]. As anticipated, ebselen oxide did not reduce the phosphorylation of gD‐HER2‐EBM*, nor downstream activation of ERK induced by gD‐HER2‐EBM* overexpression (Fig. 1H). In addition, although HER2 is the preferential heterodimerization partner of the other HER family members [13], ebselen oxide did not alter the physiological activation of HER2 in heterodimers with EGFR/HER1 or with HER3 upon EGF or HRG stimulation, nor downstream activation of AKT and ERK MAPK, in noncancer cells expressing normal levels of HER2 (Fig. S2). These results demonstrate that ebselen oxide specifically prevents the oncogenic ligand‐independent activation of HER2.

**Table 1:** Compounds structurally close to ebselen with diverse degree of inhibition of HER2/FERM interaction, HER2 activation, and anchorage‐dependent and ‐independent proliferation of HER2^+^ breast cancer cells Analog 1: 2‐(4‐methylphenyl)‐1,2‐benzisothiazol‐3(2H)‐one; Analog 2: 2‐(4‐chlorophenyl)‐1,2‐benzisothiazol‐3(2H)‐one; Analog 3: 2‐(4‐methoxyphenyl)‐1,2‐benzoselenazol‐3‐one; Analog 4: N‐phenylphtalimide; Analog 5: 2‐(4‐isopropyl phenyl)‐1‐isoindolinone; Analog 6: 2‐(4‐Methylphenyl)‐1‐oxo‐1,2‐benzothiazol‐3‐one; Analog 7: 2‐[4‐(trifluoromethyl)phenyl]‐1,2‐benzothiazol‐3‐one; Analog 8: 2‐(4‐chlorophenyl)‐1‐oxo‐1,2‐benzothiazol‐3‐one. Active compounds (binding to and inhibiting HER2) are shown in red, and inactive compounds are shown in green (binding to HER2 and no specific HER2 inhibition) or in blue (not binding to HER2). NA, nonapplicable; ND, not determined.

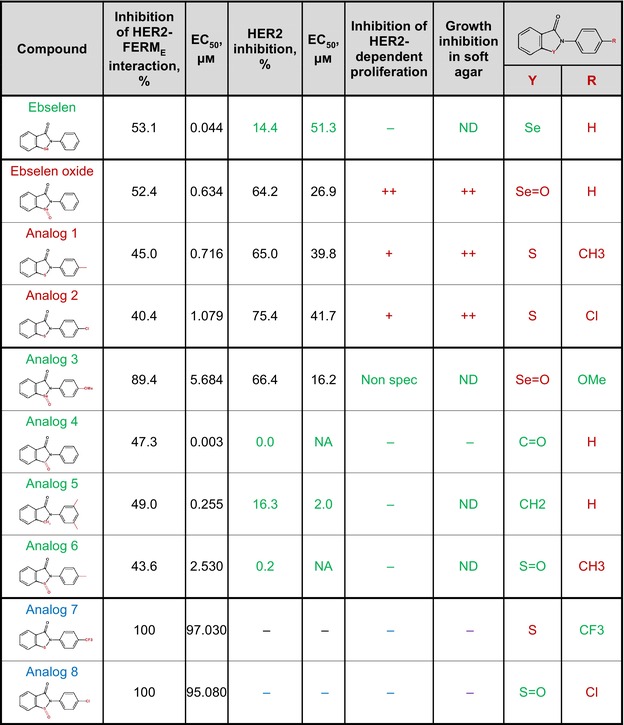

### 3.2 Ebselen oxide inhibits the proliferation of HER2^+^ cancer cells

**Fig. 2:**
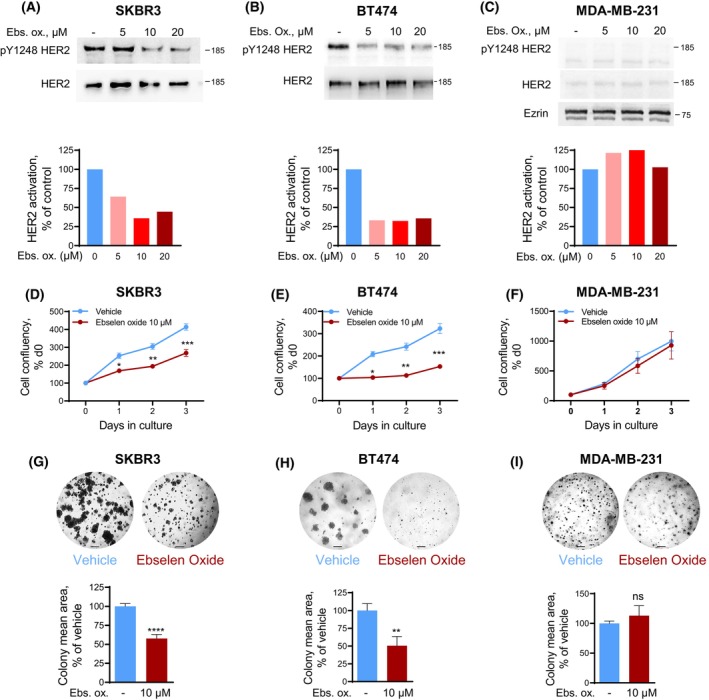
Ebselen oxide inhibits HER2 activation, cell proliferation, and anchorage‐independent growth of HER2^+^ breast cancer cells. (A–C) Lysates from HER2‐positive (SKBR3, A and BT474, B) or HER2‐negative (MDA‐MB‐231, C) breast cancer cells treated with ebselen oxide (0–20 μm) were analyzed by western blot using antibodies against pY1248 HER2, HER2 and ezrin and the optical density was quantified. Blot and quantification of a representative experiment are shown out of 4 (A), 5 (B), or 2 (C) independent experiments. (D–F) Proliferation curves of HER2‐positive (SKBR3, D or BT474, E) or HER2‐negative (MDA‐MB‐231, F) breast cancer cell lines treated with vehicle or 10 μm ebselen oxide assessed by MTT assay. A representative experiment is shown out of 5 (D), 4 (E), or 4 (F) independent experiments where data are mean ± SEM; 2‐way ANOVA (mixed‐effect analysis) followed by Bonferroni's multiple comparison test, *P < 0.05, **P < 0.01, ***P < 0.001. (G–I) Anchorage‐independent growth of SKBR3 (G), BT474 (H), or MDA‐MB‐231 (I) cells treated with vehicle or 10 μm ebselen oxide. Representative images and quantification of the colony mean area are shown where data are mean ± SEM (*n* = 2), unpaired *t* test, ****P < 0.0001 (*n* = 4–12) (G); (*n* = 2), unpaired t test, **P < 0.01 (*n* = 6–9 replicates) (H); (*n* = 2), unpaired *t* test, ^ns^ *P* = 0.4325 (*n* = 6–7 replicates) (I). Scale bars are 200 μm.

We next addressed if ebselen oxide could block HER2 activation and cell proliferation in HER2^+^ (SKBR3 and BT474) breast cancer cell lines. In SKBR3, ebselen oxide induced a dose‐dependent inhibition of HER2 reaching 66% at 24 h, (EC_50_ = 23.9 μm) that was sustained to 64% at 48 h (EC_50_ = 26.9 μm) (Table 1, Fig. 2A, and Fig. S3A). Similar results were observed in BT474 (Fig. 2B). On the contrary, ebselen oxide had no effect on HER2 activation in tumorigenic MDA‐MB‐231 breast cancer cell line exhibiting a low level of HER2 expression (Fig. 2C and Fig. S3B). Using a proliferation assay based on the cellular metabolic activity, we observed that 10 μm ebselen oxide reduced by 35–55% the proliferation rate of HER2^+^ SKBR3 or BT474 cells, respectively, while it did not reduce the proliferation rate of MDA‐MB‐231 (Fig. 2D–F). Identical results were observed with an impedance‐based real‐time proliferation assay (Fig. S3C–E). These results demonstrate that ebselen oxide specifically inhibits HER2‐dependent cell proliferation.

We then evaluated the ability of ebselen oxide to block the anchorage‐independent proliferation of breast cancer cell lines in a soft agar assay, as it allows a close mimicry of the 3D cellular environment seen *in vivo* and this gold‐standard method gives results that correlate closely with tumor progression *in vivo* [23]. In this setting, treatment with 10 μm ebselen oxide induced more than 40% and 50% reduction in the size of SKBR3 and BT474 micro‐colonies, respectively (Fig. 2G,H). On the contrary, 10 μm ebselen oxide had no impact on the development of micro‐colonies originating from MDA‐MB‐231 cells (Fig. 2I). These experiments showed that ebselen oxide specifically inhibits the anchorage‐independent proliferation of HER2^+^ breast cancer cells.

**Fig. 3:**
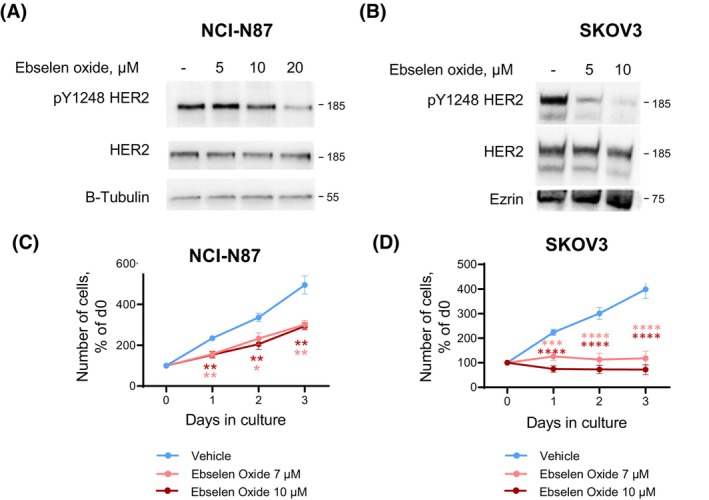
Ebselen oxide inhibits HER2 activation and HER2‐dependent proliferation in gastric and ovarian HER2^+^ cancer cells. (A) HER2‐positive NCI‐87 gastric cancer cells were treated with ebselen oxide (0–20 μm) for 24 h, lysed, and analyzed by western blot using anti‐pY1248‐HER2, HER2, and β‐tubulin antibodies. A representative western blot is shown out of 4 independent experiments. (B) HER2‐positive SKOV3 ovarian cancer cells were treated with ebselen oxide (0–10 μm) for 24 h, lysed, and analyzed by western blot using anti‐pY1248‐HER2, HER2, and ezrin antibodies. A western blot is shown (*n* = 1). (C, D) Proliferation curves of NCI‐N87 gastric (C) or SKOV3 ovarian (D) cancer cells treated with vehicle or 7–10 μm ebselen oxide measured by MTT assay. Data are mean ± SEM (*n* = 9–12) of three independent experiments. Where two‐way ANOVA followed by Dunnett's multiple comparison test, *P < 0.05, **P < 0.01, ***P < 0.001, and ****P < 0.0001.

We then investigated the effect of ebselen oxide on other HER2^+^ cancers cells using gastric (NCI‐N87) and ovarian (SKOV3) cell lines, which display high expression levels of HER2 and exhibit strong HER2‐dependent proliferation as revealed by the decrease of both HER2 activation and proliferation capacity with AG1478, a nonspecific kinase inhibitor of HER2 (Fig. S4). In both NCI‐N87 and SKOV3 cell lines, ebselen oxide also strongly blocked HER2 activation, an effect accompanied by reduced proliferation, revealing the potent action of ebselen oxide on several HER2^+^ cancers (Fig. 3).

### 3.3 Ebselen oxide inhibits activation of truncated and mutated oncogenic forms of HER2

**Fig. 4:**
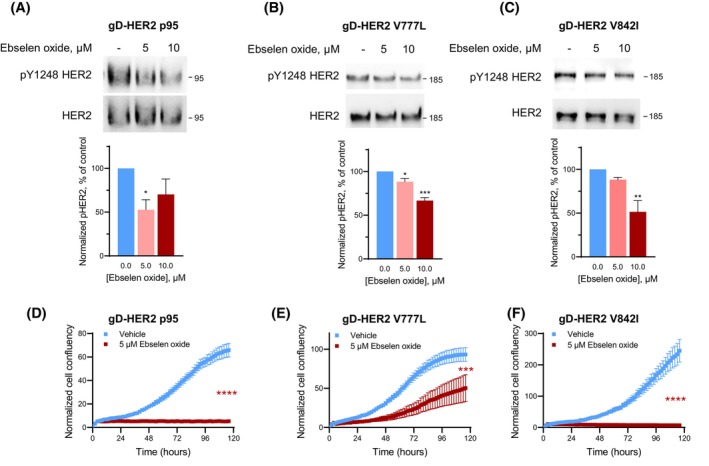
Oncogenic p95, V777L, and V842I forms of HER2 are sensitive to treatment by ebselen oxide. (A–F) HBMECs transfected with vectors encoding gD‐tagged p95 (A, D), V777L (B, E), or V842I (C, F) HER2 altered forms were treated or not during 24 h with 5–10 μm ebselen oxide. (A–C) Lysates were then analyzed by western blot using antibodies against pY1248 HER2 and HER2. Representative blot and data quantification (mean ± SEM) are shown out of 3 (A), 3 (B), or 4 (C) independent experiments. One‐way ANOVA followed by Dunnett's multiple comparison test, *P < 0.05, **P < 0.01, ***P < 0.001. (D–F) Proliferation was assessed by Incucyte during 5 days. Data are mean ± SEM of a representative experiment out of two independent experiments. Two‐way ANOVA followed by Bonferroni's multiple comparison test, ***P < 0.001, ****P < 0.0001.

We next tested the capacity of ebselen oxide to inhibit the activation of several mutated and truncated forms of HER2 associated with resistance to anti‐HER2 therapeutic agents, including the truncated form of HER2 (p95HER2), which is expressed in up to 30% of HER2^+^ tumors and presents intrinsic resistance to antibody‐based therapies [4, 5, 19] and V777L HER2 and V842I HER2, two activating somatic mutations of HER2 kinase domain found in 4.0% and 4.4% of all cancers, respectively, and associated with trastuzumab and/or resistance to kinase inhibitors [24, 25]. HBMECs cells overexpressing gD‐tagged p95HER2, V777L, or V842I HER2 were treated with ebselen oxide. 5–10 μm ebselen oxide strongly inhibited p95HER2 activation (Fig. 4A). Comparable inhibitory activity of ebselen oxide was observed toward V777L HER2 and V842I HER2 with a 34–49% inhibition of their activation (Fig. 4B,C). As a result, 5 μm ebselen oxide almost totally inhibited the cell proliferation induced by the overexpression of p95, V777L, and V842I forms of HER2 (Fig. 4D–F and Fig. S5A,B) while it did not affect the proliferation of cells transfected with pRK5 empty vector (Fig. S5A,B). The same effects were observed for V777L and V842I forms of HER2 with another proliferation assay based on metabolic activity (Fig. S5C). Compared with AG1478 (5 μm), ebselen oxide (5 μm) achieved similar (V842I) or better (V777L) inhibition of the proliferation of cells expressing HER2 mutant forms (Fig. S5C).

These results demonstrate that, by targeting the ERM‐binding motif in HER2 juxtamembrane region, ebselen oxide inhibits HER2 oncogenic forms known to confer resistance to anti‐HER2 therapies and thus provides a significant advantage over existing HER2‐targeted therapies.

### 3.4 Ebselen oxide is the most active ebselen derivative promoting HER2 inhibition

Given the promising effects of ebselen oxide, we then evaluated several structurally related compounds in order to define the structural determinants essential for HER2 binding and inhibition. As shown in Table 1, analog 1 (2‐(4‐methylphenyl)‐1,2‐benzisothiazol‐3(2H)‐one) and analog 2 (2‐(4‐chlorophenyl)‐1,2‐benzisothiazol‐3(2H)‐one) both competed with HER2/FERM interaction, inhibited HER2 activation in HER2^+^ breast cancer cells and reduced the anchorage‐dependent and ‐independent proliferation of HER2^+^ breast cancer cells, although less efficiently than ebselen oxide. Conversely, despite their similar inhibition of HER2/FERM interaction, no or low inhibition of HER2 activity and HER2‐dependent cell proliferation were detected with analog 4 (N‐phenylphtalimide), 5 (2‐(4‐isopropyl phenyl)‐1‐isoindolinone), and analog 6 (2‐(4‐Methylphenyl)‐1‐oxo‐1,2‐benzothiazol‐3‐one), whereas analog 3 (2‐(4‐methoxyphenyl)‐1,2‐benzoselenazol‐3‐one), exhibited nonspecific reduction in cell proliferation. Finally, the ability of analog 7 (2‐[4‐(trifluoromethyl)phenyl]‐1,2‐benzothiazol‐3‐one) and analog 8 (2‐(4‐chlorophenyl)‐1‐oxo‐1,2‐benzothiazol‐3‐one) to compete with HER2/FERM interaction was drastically reduced as compared to ebselen oxide (Table 1). These observations allowed us to define a pharmacophore of ebselen derivatives required for HER2 inhibition where atoms in position Y and R (shown in red in structure) can be modified as followed: Y is either a S or a Se=O and R is either H or Cl or Me but not OMe or CF_3_ (Table 1). Ebselen oxide hence emerged as the most active ebselen derivative compound, promoting the blockade of HER2 by a moesin‐mimicking mechanism, and was then selected for *in vivo* evaluation.

### 3.5 Ebselen oxide blocks the progression of HER2‐positive breast tumors *in vivo*

**Fig. 5:**
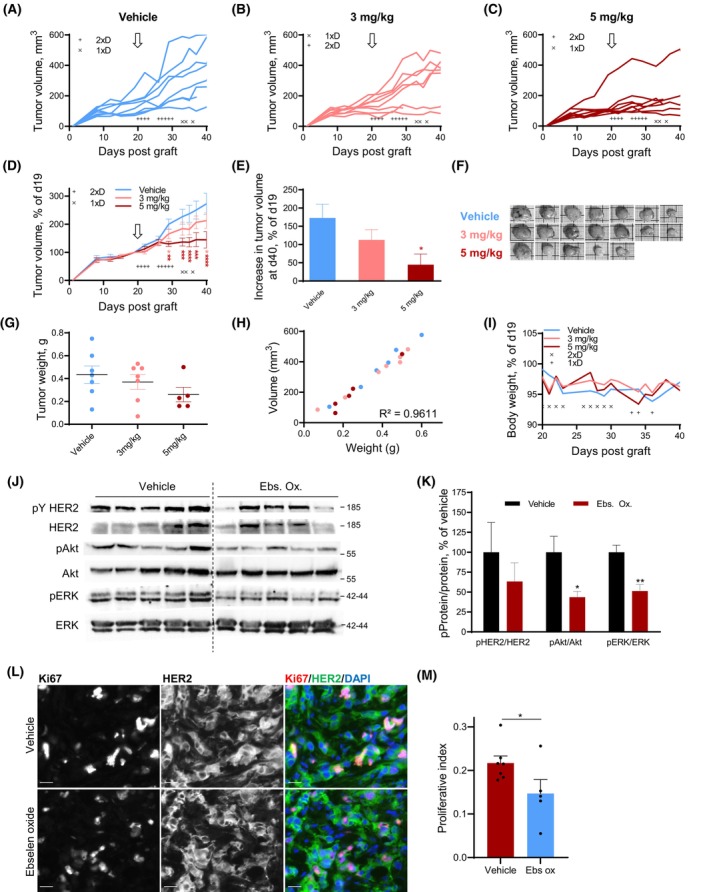
Ebselen oxide blocks the progression of HER2‐positive breast tumors *in vivo*. (A–L) 5 × 10^6^ BT474 cells were orthotopically implanted in the mammary fat pad of NOD.Cg‐Prkdc scid/J mice, together with estradiol supplement. After 19 days, mice were randomized into three groups (*n* = 8 mice per group) and administered intraperitoneally with ebselen oxide (3 or 5 mg·kg^−1^) once (1 × D) or twice (2 × D) a day, as indicated for 5 days a week or vehicle (10% DMSO in PBS) during 3 weeks. (A–C) Individual tumor volume monitoring of vehicle‐ or ebselen oxide‐treated mice, as specified. Treatment schedule is indicated by × (once a day) or + (twice a day). The beginning of the treatment is indicated by an arrow. (D) Mean tumor volume of vehicle‐ or ebselen oxide‐treated mice, normalized to the initial volume at day 19. Data are mean ± SEM; two‐way ANOVA (mixed‐effect analysis) followed by Dunnett's multiple comparison test, *P < 0.05, **P < 0.01, ***P < 0.001, ****P < 0.0001. The beginning of the treatment is indicated by an arrow. (E) Increase in tumor volume at day 40 compared with day 19 for each condition. Data are mean ± SEM; one‐way ANOVA followed by Dunnett's multiple comparison test, *P < 0.05. (F) Pictures of tumors collected at the end of the experiment. (G) Scatter plot of final tumor weight displaying mean ± SEM; one‐way ANOVA followed by Bonferroni's multiple comparison test, *P* = 0.2682. (H) Analysis of the correlation between individual tumor volume and tumor weight. Pearson correlation analysis, *r* ^2^ = 0.9611. (I) Mean mice weight monitored 3 times a week during the treatment. Treatment schedule is indicated by × (once a day) or + (twice a day). (J) Western blot analysis of the tumor lysates from vehicle‐ or ebselen oxide‐treated mice (5 mg·kg^−1^) (*n* = 5 mice per group) using antibodies directed against activated HER2 (pY HER2), HER2, activated Akt (pAkt), Akt, activated ERK (pERK), ERK. (K) Quantification of the results presented in G where data are mean ± SEM (*n* = 5), unpaired *t* test *P < 0.05, **P < 0.01. (L) Representative images of tumor sections from vehicle‐ or ebselen oxide‐treated mice (5 mg·kg^−1^) immunolabeled with anti‐Ki67 (red) and anti‐HER2 (green) antibodies and counterstained with DAPI (blue). Scale bar is 20 μm. (M) Quantification of the proliferation index in sections from vehicle‐ or ebselen oxide‐treated mice (5 mg·kg^−1^) immunolabeled with anti‐Ki67 and anti‐HER2 antibodies and counterstained with DAPI where data are mean ± SEM (vehicle *n* = 7; ebselen oxide *n* = 5), unpaired *t* test *P < 0.05.

We then explored the potential of ebselen oxide to reduce the growth of HER2‐overexpressing tumors *in vivo*. Nineteen days after orthotopic implantation of BT474 cells in the mammary fat pad of immunodeficient NOG mice, mice received 3 or 5 mg·kg^−1^ ebselen oxide, intraperitoneally (once or twice a day, as indicated, 5 days a week) or vehicle as a control. Between day 19 (beginning of the treatment) and day 40 (end of the experiment), in control animals, and to a lesser extent in animals administered with 3 mg·kg^−1^ ebselen oxide, tumor size increased by 172% and by 112%, respectively (Fig. 5A,B,D,E). Conversely, in animals treated with 5 mg·kg^−1^ ebselen oxide, it increased only by 44% in mice treated with 5 mg·kg^−1^ ebselen oxide, corresponding to a significant growth inhibition index of 75% at day 40 (Fig. 5C–E). Accordingly, while tumors collected at day 40 from mice treated with 3 mg·kg^−1^ ebselen oxide exhibited only a little reduction in their size and weight as compared to tumors from the vehicle‐treated group, tumors from mice treated with 5 mg·kg^−1^ ebselen oxide were macroscopically smaller (Fig. 5F) and displayed a 40% drop in weight as compared to the vehicle‐treated group (Fig. 5G). Of note, there was an excellent correlation between tumor volume and tumor weight (*R* ^2^ = 0.9611, Fig. 5H). Both dosages of ebselen oxide were well‐tolerated until day 30 as shown with the little variation of mice body weight (Fig. 5I). However, a decrease of mice body weight in ebselen oxide‐treated animals after day 30 prompted us to alleviate the treatment from two to one injection per day, after which mice rapidly recovered. We next analyzed biochemically the intratumoral level of activated HER2 and downstream signaling in vehicle‐ and 5 mg·kg^−1^ ebselen oxide‐treated mice. In tumor lysates from ebselen oxide‐treated animals, HER2 activation was decreased by 30% compared with vehicle‐treated animals (Fig. 5J,K) accompanied by a 50% inhibition of Akt activation and 45% inhibition of ERK activation (Fig. 5J,K). Additionally, immunofluorescence analysis on tumor sections using anti‐Ki67 antibody revealed that ebselen oxide was associated with a 32% drop of HER2^+^ breast tumor proliferation index compared with vehicle‐treated mice (Fig. 5L,M). In conclusion, these results strongly support a robust HER2 inhibitory effect of ebselen oxide on human breast cancer cells overexpressing HER2 *in vivo*.

### 3.6 Additive effect of known anti‐HER2 agent and ebselen oxide

**Fig. 6:**
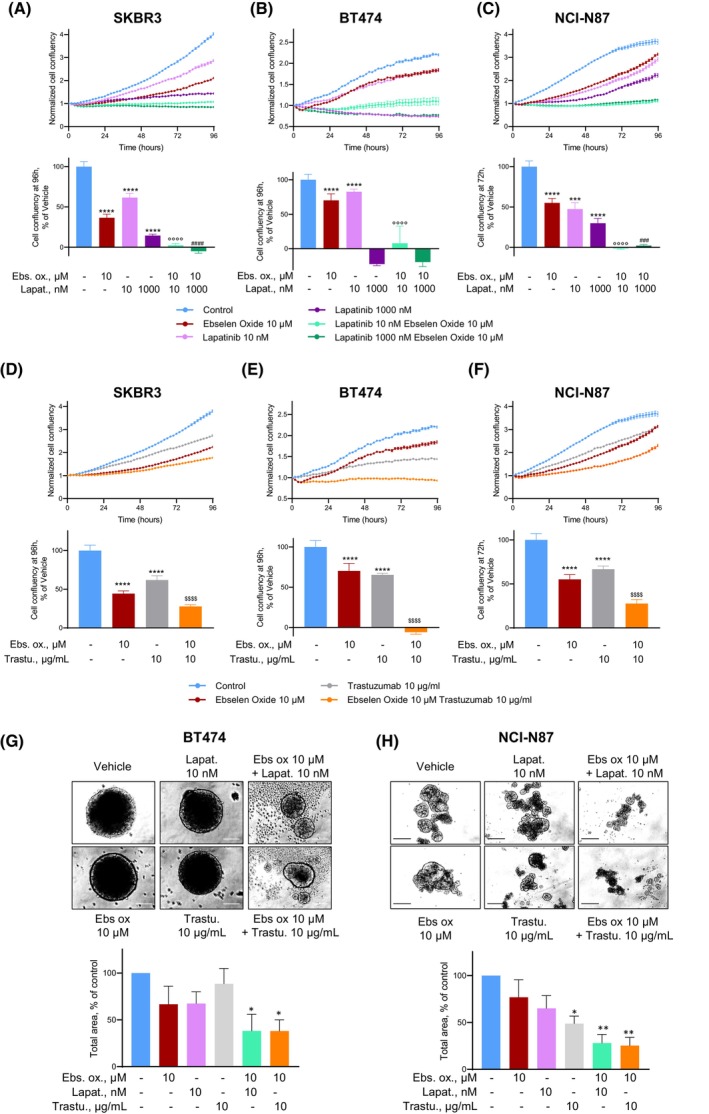
Additive effects of known anti‐HER2 agent and ebselen oxide. (A–C) Proliferation curves of HER2‐positive (SKBR3, A; BT474, B; NCI‐N87, C) cancer cell lines treated or not with ebselen oxide (10 μm), lapatinib (10–1000 nm), or combination of these agents, as indicated. Proliferation was assessed overtime by Incucyte during 4 days. Data are mean ± SD (*n* = 8–12) of a representative experiment out of 4 (A), 3 (B), or 2 (C) independent experiments. One‐way ANOVA followed by Sidak multiple comparison test, (*, compared with Vehicle; ° and #, respectively, compared with lapatinib 10 and 1000 nm) ***P < 0.001, ****P < 0.0001. (D‐F) Proliferation curves of HER2‐positive (SKBR3, D; BT474, E; NCI‐N87, F) cancer cell lines treated or not with ebselen oxide (10 μm), trastuzumab (10 μg·mL^−1^), or combination of these agents, as indicated. Proliferation was assessed by Incucyte during 4 days. Data are mean ± SD (*n* = 8–12) of a representative experiment out of two independent experiments. One‐way ANOVA followed by Sidak multiple comparison test, (*, compared with Vehicle; $, compared with trastuzumab) ***P < 0.001, ****P < 0.0001. (G, H) Anchorage‐independent growth of HER2‐positive (BT474, G or NCI‐N87, H) cancer cells treated or not ebselen oxide (10 μm), lapatinib (10 nm), trastuzumab (10 μg·mL^−1^), or a combination of these agents, as indicated. Images of a representative experiment out of 3 (G) or 4 (H) independent experiments and quantification of the colony total area are shown where data are mean ± SEM. One‐way ANOVA followed by multiple comparison test, *P < 0.05, **P < 0.01. Scale bars are 100 μm.

Because they target completely different regions of the HER2 receptor, we addressed a potential benefit of the association between ebselen oxide and standard anti‐HER2 therapeutic agents in HER2‐positive cancer cells, from breast (SKBR3 and BT474) or gastric (NCI‐N87) origin. Regarding the combination with HER2 kinase inhibitor, we found that although 10 μm ebselen oxide or 10 nm lapatinib partially decreased HER2 activation, combination of both totally abolished HER2 activation in SKBR3 and NCI‐N87 HER2‐overexpressing cell lines (Fig. S6A,B). In agreement, while SKBR3 breast cancer cell proliferation was reduced by 39% after 10 nm lapatinib or by 64% using 10 μm ebselen oxide, combination of both agents achieved total inhibition of SKBR3 proliferation and was thus far more effective than lapatinib alone even when used up to 1 μm (Fig. 6A). The same was observed in BT474 and NCI‐N87 cancer cells, in which combination of 10 nm lapatinib and 10 μm ebselen oxide completely inhibited cell proliferation compared with partial effects of agents when used separately [18% (BT474) or 53% (NCI‐N87) reduction with 10 nm lapatinib and 30% (BT474) or 45% (NCI‐N87) reduction with 10 μm ebselen oxide] (Fig. 6B,C). Noteworthy, in SKBR3 and NCI‐N87 cells, combination of 10 μm ebselen oxide and 10 nm lapatinib was more effective than lapatinib alone at a dose 100 times higher.

Regarding combination with antibody‐based therapies, trastuzumab (10 μg·mL^−1^) induced a strong decrease of HER2 activation, which was potentiated by the addition of ebselen oxide (Fig. S6C). Similar benefits of trastuzumab/ebselen oxide combination were observed in both breast and gastric cancer cell lines with 75% (SKBR3 and NCI‐N87) to complete (BT474) inhibition of cell proliferation compared with 10 μg·mL^−1^ trastuzumab alone [35% (BT474), 38% (SKBR3), or 34% (NCI‐N87)] or to 10 μm ebselen oxide alone [30% (BT474), 64% (SKBR3), and 45 (NCI‐N87)] (Fig. 6D–F). Accordingly, ebselen oxide combined with either lapatinib or trastuzumab was more efficient to reduce the anchorage‐independent growth (spheroid assay) of BT474 than each drug administered separately (Fig. 6G). The same observation was made in NCI‐N87 [62% (ebs ox/lapatinib or 65% (ebs ox/trastuzumab) compared with 23% (ebs ox), 35% (lapatinib), or 12% (trastuzumab))] (Fig. 6H). Importantly, as shown for ebselen oxide alone (Fig. 2C,F,I) the combination of these treatments had no effect on the proliferation of non‐HER2‐overexpressing cancer cells such as MDA‐MB‐231 (Fig. S6D).

In conclusion, combining ebselen oxide with anti‐HER2 agents, lapatinib or trastuzumab, could provide a strong benefit as these combined treatments reached greater efficacy than treatment with single molecules to block HER2 signaling in HER2‐positive cancer cells and would thus allow the use of sub‐optimal doses and subsequent reduction of toxicity‐related adverse effects.

## 4 Discussion

Over the last years, although considerable efforts have been made in the treatment of HER2^+^ breast cancer, the emergence of resistance mechanisms to HER2‐targeted therapies, as well as their adverse effects stressed the need for the identification of new drugs acting on mutated and truncated forms of the receptor. In this work, we identified a novel class of anti‐HER2 inhibitors targeting the cytosolic juxtamembrane region of HER2. Ebselen oxide demonstrated a potent anti‐HER2 activity resulting in a strong inhibition of both anchorage‐dependent and ‐independent proliferation of HER2‐positive cancer cells. Importantly, ebselen oxide blocked HER2^+^ breast tumor progression in an orthotopic xenograft model and had a strong action on mutated or truncated forms of HER2.

Previous works have demonstrated that the juxtamembrane region plays a crucial role in the activation of HER receptors upon ligand binding, as it stabilizes the formation of asymmetric kinase domain dimers allowing the transphosphorylation process [26, 27, 28]. We then demonstrated that, unlike other members of the receptor tyrosine kinase family, HER2 possesses in its cytosolic juxtamembrane domain a motif allowing interaction with ERM proteins [14]. Despite the unique structural conformation of the extracellular domain of HER2 locked in an open and active conformation, this interaction maintains HER2 in a catalytically repressed state, most likely by exerting a constraint on the juxtamembrane region preventing stabilization in activated dimers. In cancer cells, the concomitant overexpression of HER2 and loss of moesin expression contributes to HER2 activation, which can be reverted with the use of moesin‐mimicking compounds [14]. In this work, we clearly demonstrated that ebselen oxide behaves as a moesin‐mimicking compound, providing an allosteric inhibition of HER2 through binding to the juxtamembrane region of HER2. We showed that ebselen oxide competes with ezrin for binding to this region and directly binds to the juxtamembrane region of HER2. Moreover, its ability to inhibit HER2 but not a mutant in the juxtamembrane domain of HER2, which is no longer able to interact with ERM, confirms the targeting of this region. We also unambiguously established that ebselen oxide selectively inhibits HER2 in HER2‐overexpressing cells, whereas no effect was reported in cells that display a low level of HER2 (MDA‐MB‐231 or HBMECs). Finally, because the ERM‐binding motif is absent from the other HER family member, we showed that ebselen oxide does not affect the ligand‐dependent activation of HER2 in heterodimers with the other family members. This mechanism of action might offer several benefits to ebselen oxide as compared to current anti‐HER2 therapies that are nonselective and also target heterodimeric HER2 activation [29]. Indeed, since the ligand‐dependent activation of HER2 plays an essential role in cardiac development and physiological function of the adult heart, the current anti‐HER2 therapies induce significant cardiotoxicities [11, 30], as well as high incidence of diarrhea, nausea, and skin rashes due to the inhibition of EGFR/HER1 function in epithelial tissues such as skin and mucosa [31, 32]. Because ebselen oxide selectively blocks the ligand‐independent activation of HER2, it is expected that it would not induce such adverse effects.

Compared with other clinically used HER2 targeting existing agents, ebselen oxide achieved similar or better efficacy than low doses of lapatinib or trastuzumab. Moreover, we unambiguously demonstrated that ebselen oxide in combination with lapatinib or trastuzumab exerts an additive effect on the inhibition of HER2 activation and/or proliferation of HER2‐positive cancer cells. Noteworthy, using spheroid assays, which replicate several key features of solid tumors and are recognized as useful tools to investigate several anticancer treatments [33], we further confirmed that combining ebselen oxide with anti‐HER2 agents could provide a strong benefit as these combined treatments reached greater efficacy than molecules administrated separately to block HER2 signaling in HER2‐positive cancer cells. Combined treatments could then allow the use of sub‐optimal doses and subsequent reduction of toxicity‐related adverse effects, promising ebselen oxide as an effective adjuvant treatment of HER2‐positive cancers.

We further showed that ebselen oxide is active on several intrinsically altered forms of HER2 such as the p95 truncated form of HER2, which is expressed in up to 30% of patients and confers resistance to antibodies‐based therapies and is thus associated with poor outcomes in trastuzumab‐treated HER2‐positive metastatic breast cancer [4, 5, 19]. Other HER2 mutations have been described to induce resistance to actual treatments as they favor HER2 dimerization, increase the stability of the receptor, or alter interaction with regulatory partners. As a consequence, those are linked to the worse patient outcome [34]. We showed that ebselen oxide can suppress the activity of HER2 harboring V777L or V842I mutations that were found, respectively, in 4.0% and 4.4% of all cancers [25] and, due to its mechanism of action, it is also expected to act on other HER2 alterations. Finally, HER2 is aberrantly expressed in a wide range of malignancies including gastric, ovarian, bladder, salivary gland, endometrial, pancreatic, and non‐small‐cell lung cancers, and the benefit of anti‐HER2 therapies is either evaluated or has already been demonstrated [35, 36]. As anticipated, we showed that ebselen oxide exerts antitumor effects on HER2^+^ gastric and ovarian carcinoma cell lines. Given this effect, it is expected that it would be active on all HER2^+^ cancers.

In the course of these experiments, we found that, whereas ebselen did not inhibit HER2, its selenoxide analog had potent anti‐HER2 activity. Interestingly, ebselen is a synthetic organoselenium drug molecule with anti‐inflammatory, antioxidant, and cytoprotective activity. It is a potent scavenger of hydrogen peroxide and can also react with peroxynitrite to form ebselen oxide. *In vivo* ebselen rapidly reacts with peroxynitrite [22]. Hence, it is of particular interest to observe an efficient inhibition of HER2 with its oxidative product, devoid of antioxidant properties, and which acts through direct interaction with HER2. In an effort to discover and optimize the effects observed with ebselen oxide, we also evaluated several compounds structurally close to this compound and identified two other drugs, analogs 1 and 2, where the selenium is replaced by a sulfur atom, displaying HER2 inhibitory action almost comparable to that of Ebselen oxide. However, the oxidation of the sulfur seems to result in a loss of the activity toward HER2 inhibition (analogs 6 and 8). Analogs 4 and 5 demonstrated a greater binding affinity for the cytosolic juxtamembrane region of HER2 as compared to ebselen oxide, but they did not exert any HER2 inhibitory effect suggesting that binding of the drugs to HER2 is necessary but not sufficient to promote HER2 inhibition.

**Fig. 7:**
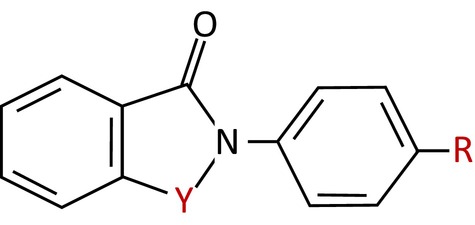
Pharmacophore of Ebselen‐like HER2 inhibitors. Structure of the defined pharmacophore of ebselen‐like HER2 inhibitors, where atoms in position Y can be either selenium (S) or selenium oxide (Se=O), and either hydrogen (H), chlorine (Cl), or methyl (CH3) but not methoxy group (OMe) nor trifluoromethyl (CF3) on position R.

Overall, in this work we described a family of allosteric inhibitors of HER2 with a detailed knowledge of the pharmacophore, where atoms in position Y and R can be either selenium (S) or selenium oxide (Se=O) on position Y and either hydrogen (H), chlorine (Cl), or methyl (CH3) but not a methoxy group (OMe) nor trifluoromethyl (CF3) on position R (Fig. 7). Interestingly, selenium‐containing molecules have been shown to exert antiproliferative and proapoptotic effects on a wide range of cancer cell types and to potentiate radiotherapy efficacy [37]. Several selenium‐containing compounds have also been reported to inhibit key cancer targets, among which several kinases [37]. We describe here a novel antitumoral effect of a selenium‐containing compound, which acts independently of antioxidant activity, through direct interaction with a major oncogene driving the development of a variety of cancer types.

### 4.1 Ebselen oxide from bench to bedside?

Given its innovative mode of action, it is tempting to propose the use of ebselen oxide for the treatment of HER2^+^ cancers. To our knowledge, ebselen oxide has not been evaluated in humans. However, ebselen has been investigated as a possible treatment for reperfusion injury and stroke, hearing loss, tinnitus, and bipolar disorder [38]. Noteworthy, ebselen is highly active in inhibiting the main protease of SARS‐CoV‐2 and has been evaluated in phase 2 clinical trial for its ability to combat SARS‐CoV‐2 infection in moderate, as well as severely affected COVID‐19 patients [39]. Pharmacokinetics studies revealed that maximum ebselen serum concentrations were reached 1 h after intravenous injection with a half‐life of 1–3 h [40]. Due to the short half‐life, patients orally received up to 600 mg ebselen twice daily, [41]. Ebselen proved to be always well tolerated *in vivo*, exerting no sign of toxicity whatever the administered doses [38]. Considering that ebselen might rapidly form ebselen oxide *in vivo*, in our mouse model, we observed the antitumor effect of ebselen oxide at a dose regimen that might be compatible with human use. In these experiments, we detected a significant 40% decrease in the mammary tumor volume upon ebselen oxide administration that was perfectly correlated with tumor weight and similar to what we described with zuclopenthixol, another moesin‐mimicking compound we identified [14]. As we demonstrated that loss of moesin is correlated with increased HER2 expression in multiple cancers, the use of the moesin‐mimicking compound to inhibit HER2 such as ebselen oxide could represent a personalized therapeutic option for HER2^+^ cancer patients displaying a low intratumoral level of moesin.

Moreover, a major complication of HER2‐positive cancers remains the rapid progression of the disease to the brain leading to the formation of brain metastases in up to 50% of patients treated for HER2^+^ breast cancers [42]. Actual therapeutic options to treat these brain metastases are very limited due to the poor penetration of therapeutic agents across the blood–brain barrier. Ebselen has been shown to be blood–brain barrier permeant [43], and since ebselen oxide is structurally very close to ebselen, it is expected to have similar properties. Ebselen oxide would then provide a valuable benefit for the treatment of HER2^+^ breast cancers and other HER2^+^ tumors in combination with the actual HER2‐targeted therapeutic strategies.

## 5 Conclusions

Taken together, we identified ebselen oxide as a moesin‐mimicking compound illustrating a novel approach to target oncogenic forms of HER2, including mutated and truncated forms of HER2 that are resistant to actual HER2‐targeted therapies. We made the proof of concept that ebselen oxide possesses strong antitumor activity in HER2^+^ cancers alone and in combination with current treatment. Ebselen oxide might then represent a novel therapeutic option to overcome the resistance to anti‐HER2 therapy in HER2‐overexpressing cancers.

## Conflict of interest

The authors declare no conflict of interest.

## Author contributions

CF and SB conceived the study and designed the experiments. CF, LB, and HBS conducted the experiments. All authors analyzed and interpreted the data. CF and SB wrote the manuscript. All authors contributed to manuscript revisions.

### Peer review

The peer review history for this article is available at https://www.webofscience.com/api/gateway/wos/peer‐review/10.1002/1878‐0261.13419.

## Supporting information


**Fig. S1.** Ebselen does not block HER2 activation.
**Fig. S2.** Ebselen oxide does not block HER2 ligand‐dependent activation in heterodimers with the other HER family members.
**Fig. S3.** Ebselen oxide inhibits HER2 activation and proliferation of HER2^+^ breast cancer cell.
**Fig. S4.** NCI‐N87 and SKOV3 cancer cell lines are addicted to the HER2 pathway.
**Fig. S5.** Ebselen oxide inhibits the cell proliferation induced by WT and mutant forms of gD‐HER2.
**Fig. S6.** Ebselen oxide potentiates HER2 inhibition induced by anti‐HER2 agents in HER2‐positive but not in HER2‐negative breast cancer cells.

## Data Availability

The data that support the findings of this study are available from the corresponding author upon reasonable request.
