# Supplementary material for: Ebselen oxide and derivatives are new allosteric HER2 inhibitors for HER2‐positive cancers
Source: Mol Oncol. 2023 Mar 27;17(10):1981–99. doi: 10.1002/1878-0261.13419 (PMC10552892; doi:10.1002/1878-0261.13419)
Supplement: Supplementary file 1 — Fig. S1. Ebselen does not block HER2 activation. Fig. S2. Ebselen oxide does not block HER2 ligand‐dependent activation in heterodimers with the other HER family members. Fig. S3. Ebselen oxide inhibits HER2 activation and proliferation of HER2+ breast cancer cell. Fig. S4. NCI‐N87 and SKOV3 cancer cell lines are addicted to the HER2 pathway. Fig. S5. Ebselen oxide inhibits the cell proliferation induced by WT and mutant forms of gD‐HER2. Fig. S6. Ebselen oxide potentiates HER2 inhibition induced by anti‐HER2 agents in HER2‐positive but not in HER2‐negative breast cancer cells. [file MOL2-17-1981-s001.pdf]

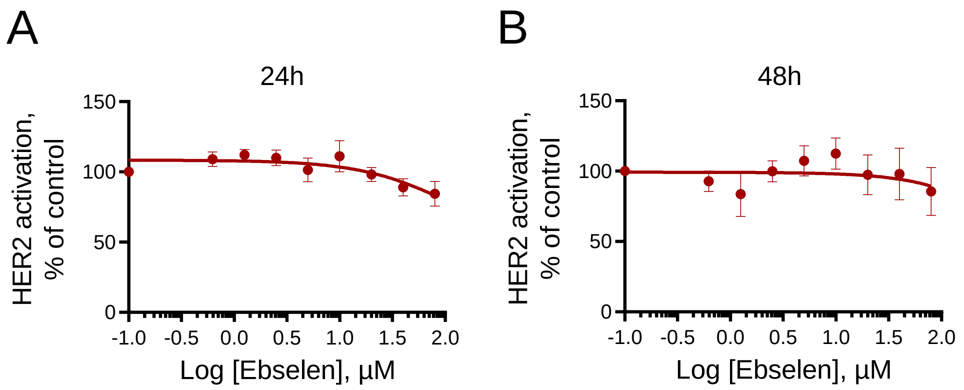

Supplementary figure 1 : Ebselen does not block HER2 activation

(A-B) Quantification of HER2 activation (pY1248-HER2) in HER2+ breast cancer cells (SKBR3) treated with increasing concentrations of Ebselen (0-80 µM) for 24h (A) or 48h (B) measured by dot blots.

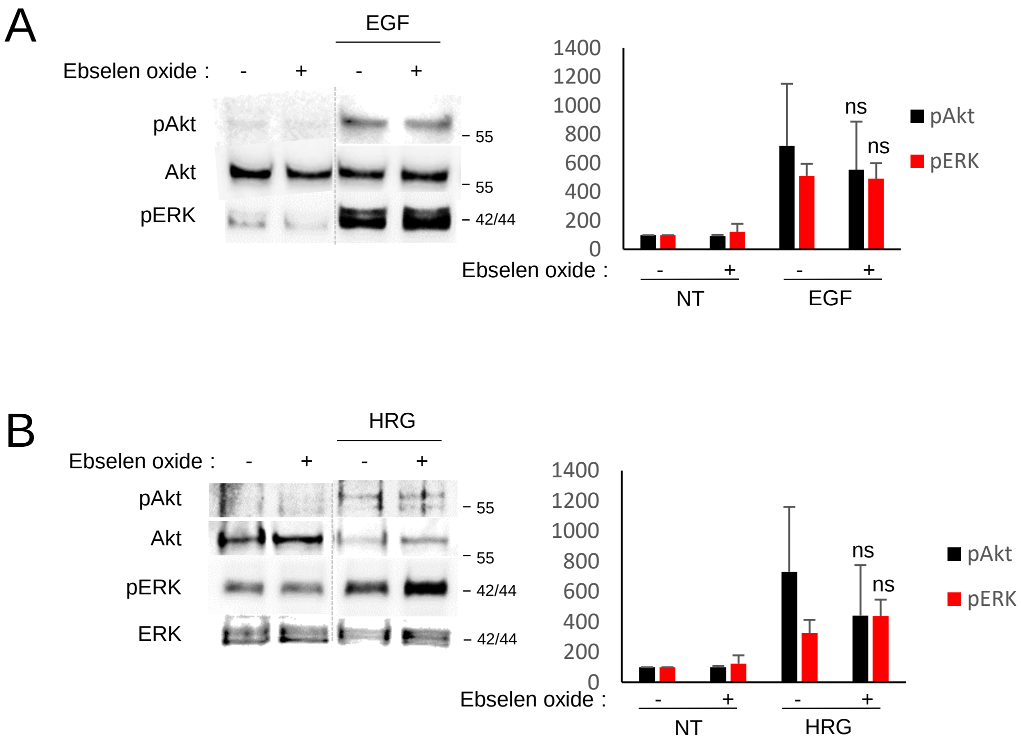

Supplementary figure 2 : Ebselen oxide does not block HER2 ligand-dependent activation in heterodimers with the other HER family members.

(A) HBMECs were treated with or without 10 µM Ebselen oxide before 5 min stimulation with 50 ng/ml EGF. Whole cell lysates were analysed by western blot using anti-pAkt, Akt and pERK antibodies. Data are mean ± sem (n=3); 2-way anova followed by Bonferroni's multiple comparison test. Comparison of Ebselen oxide/EGF-treated cells to EGF-stimulated cells is shown, (ns, not significant).

(B) HBMECs were treated with or without 10 µM Ebselen oxide before 5 min stimulation with 100 ng/ml Heregulin-1β (HRG). Whole cell lysates were analysed by western blot using anti-pAkt, Akt, pERK and ERK antibodies. Data are mean ± sem (n=3). Comparison of Ebselen oxide/HRG-treated cells to HRG-stimulated cells is shown, (ns, not significant).

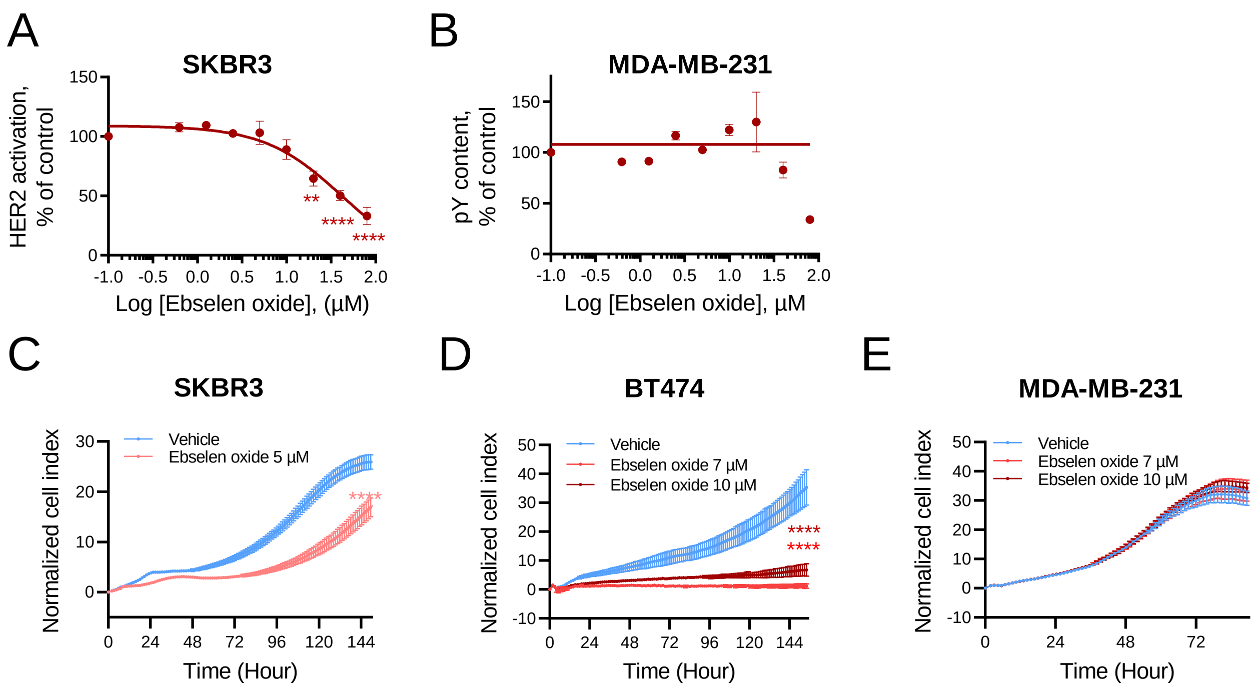

Supplementary figure 3: Ebselen oxide inhibits HER2 activation and proliferation of HER2+ breast cancer cell

(A-B) Quantification of HER2 activation (pY1248-HER2) in HER2+ (SKBR3, A) or in HER2-negative (MDA-MB-231, B) breast cancer cells treated with increasing concentrations of Ebselen oxide (0-80 µM) measured by dot blots. Data are mean ± sem (n=3); statistical analysis is Two-way ANOVA followed by multiple comparison test, **** P<0.0001, ** P<0.01

(C-E) Monitoring of the proliferation by real time impedance-based cell assay of HER2-positive SKBR3 (C) and BT474 (D) or HER2-negative MDA-MB-231 (E) breast cancer cell lines treated with vehicle or with 5-10 µM Ebselen oxide, as indicated. A representative experiment is shown (n=3-4) where data are mean ± SD (n=2-5); statistical analysis are paired t test or Two-way ANOVA followed by Dunnett's multiple comparison test, **** P<0.0001.

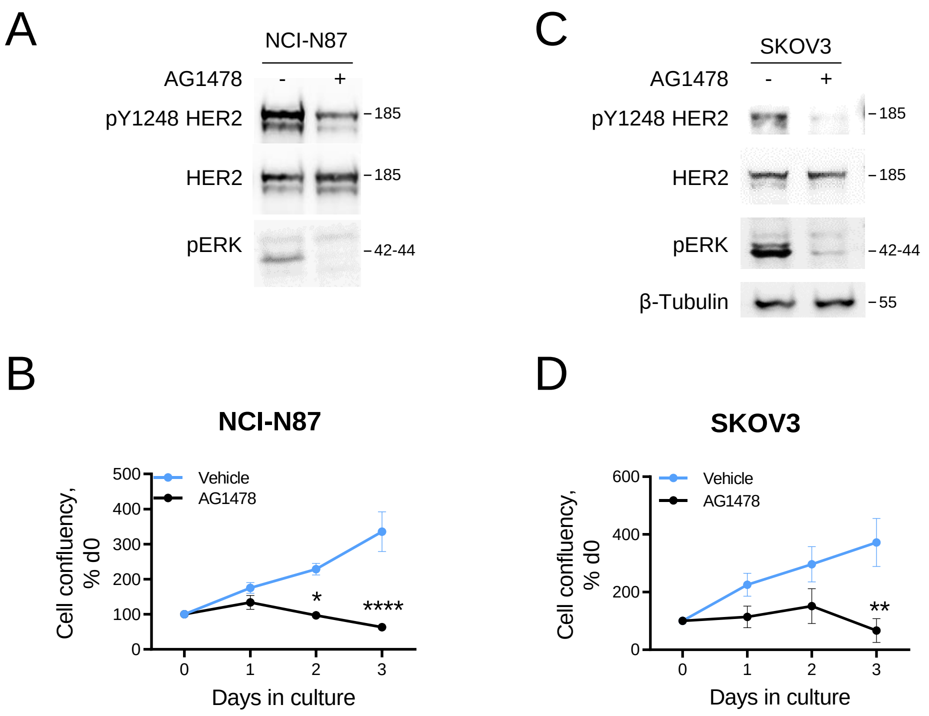

Supplementary figure 4: NCI-N87 and SKOV3 cancer cell lines are addicted to the HER2 pathway

(A) NCI-N87 cells were treated (+) or not (-) with a non-specific HER2 inhibitor AG1478 (5 µM). Whole cell lysates were analyzed by western blot with pY1248 HER2, HER2 and pERK antibodies.

(B) SKOV3 cells were treated (+) or not (-) with a non-specific HER2 inhibitor AG1478 (5 µM). Whole cell lysates were analyzed by western blot with pY1248 HER2, HER2, pERK or beta-tubulin antibodies.

(C) Proliferation curves of NCI-N87 treated or not with AG1478 (5 µM). Data are mean ± sem (n=4-6). 2-way Anova (mixed effect analysis) followed by Bonferroni's multiple comparison test, * P<0.05, **** P<0,0001.

(D) Proliferation curves of SKOV3 cells treated or not with AG1478 (5 µM). Data are mean ± sem (n=4). 2-way Anova (mixed effect analysis) followed by Bonferroni's multiple comparison test, ** P<0,01.

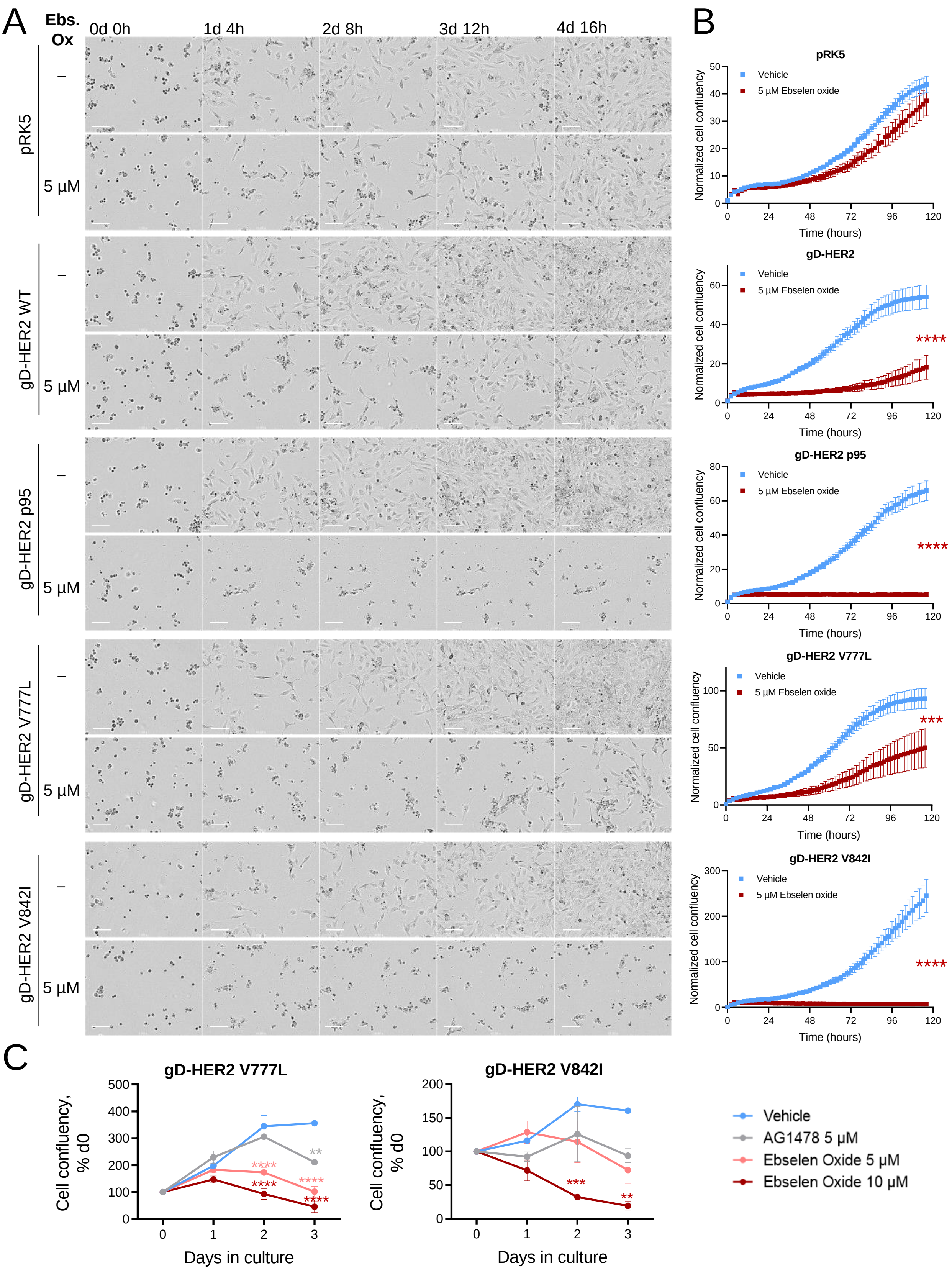

Supplementary figure 5: Ebselen oxide inhibits the cell proliferation induced by WT and mutant forms of gD-HER2

(A-B) HBMECs transfected with empty vector (pRK5) or vectors encoding gD-HER2 WT, the truncated gD-HER2 p95, or the mutated gD-HER2-V777L or V842I forms were treated or not with 5 µM Ebselen oxide. Proliferation was assessed by Incucyte during 5 days. Representative micrographs are shown (A). Representative quantification of the proliferation (B) is shown where data are mean ± sem (n=8-12). Two-way ANOVA followed by Bonferroni's multiple comparison test, *** P<0.001, **** P<0.0001. Scale bars are 100 µM.

(C) HBMECs transfected with vector encoding V777L or V842I HER2 mutants, as indicated, were treated with vehicle, 5-10 µM Ebselen oxide or 5 µM AG1478. Proliferation was assessed by MTT assay during 3 days. Data are mean ± sem (n=3). Two-way ANOVA followed by Bonferroni's multiple comparison test, * P<0.05 ** P<0.01 *** P<0.001 and**** P<0.0001.

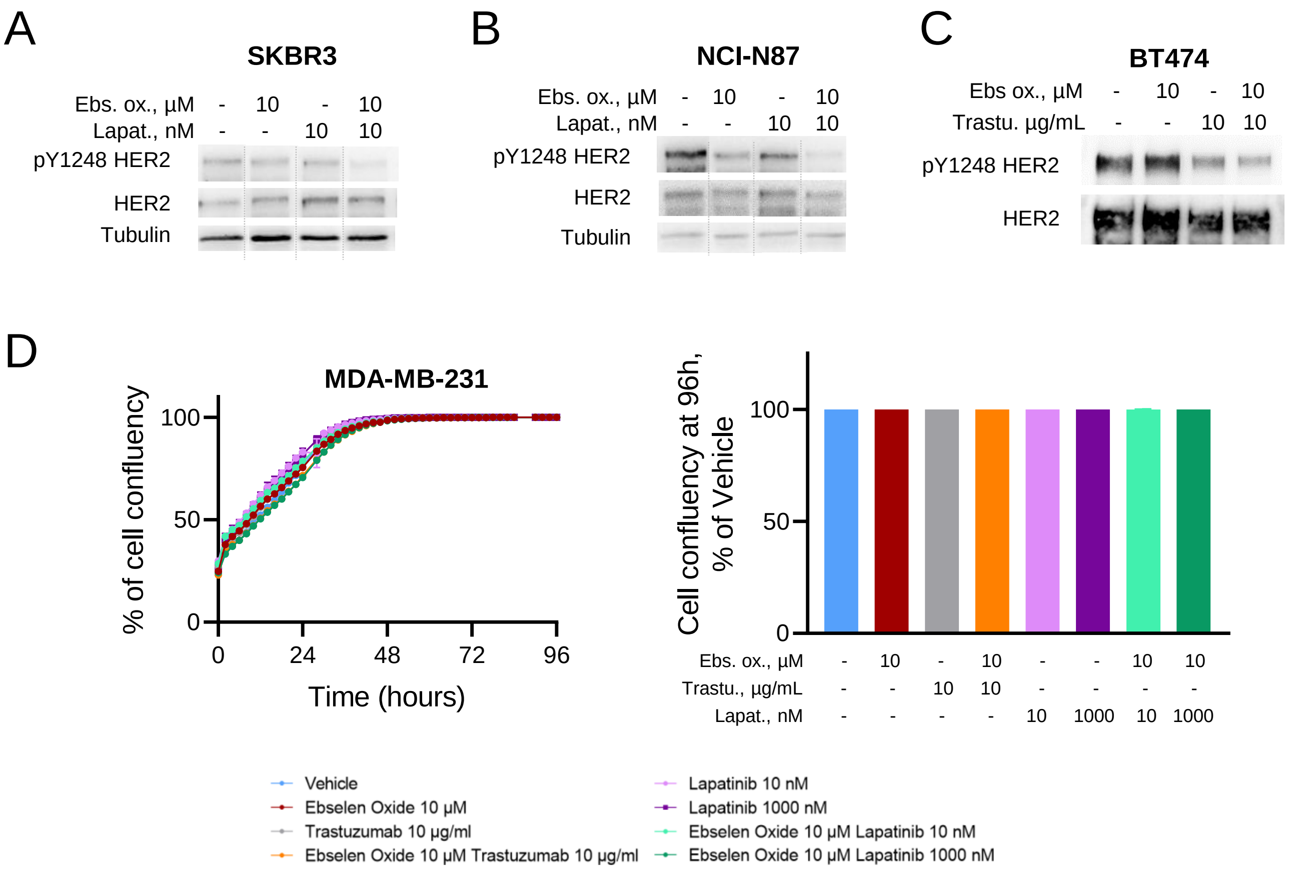

Supplementary figure 6: Ebselen oxide potentiates HER2 inhibition induced by anti-HER2 agents in HER2-positive but not in HER2-negative breast cancer cells

(A-C) HER2-positive breast cancer cells (SKBR3, A; NCI-N87, B; BT474, C) treated or not with Ebselen oxide (10 µM), lapatinib (10-1000 nM), trastuzumab (10 µg/mL) or combination of these agents, as indicated, were analyzed by western blot to evaluate HER2 activation status using antibodies directed against pY1248 HER2 and HER2.

(D) Proliferation curve of HER2-negative (MDA-MB-231) breast cancer cell lines treated or not with Ebselen oxide (10 µM), lapatinib (0.01-1 µM), trastuzumab (10 µg/mL) or combination of these agents, as indicated. Proliferation was assessed by Incucyte during 4 days. Representative curves and quantification are shown (n=2-3) where data are mean ± SD (n=8-12).
